# Human disease genomics: from variants to biology

**DOI:** 10.1186/s13059-017-1160-z

**Published:** 2017-01-30

**Authors:** Mark I. McCarthy, Daniel G. MacArthur

**Affiliations:** 1 Oxford Centre for Diabetes, Endocrinology and Metabolism, University of Oxford, Churchill Hospital, Old Road, Headington, Oxford, OX3 7LJ UK; 2 Wellcome Trust Centre for Human Genetics, University of Oxford, Roosevelt Drive, Oxford, OX3 7BN UK; 3 Oxford NIHR Biomedical Research Centre, Churchill Hospital, Old Road, Headington, Oxford, OX3 7LJ UK; 4 Program in Medical and Population Genetics, Broad Institute of MIT and Harvard, Cambridge, MA 02142 USA; 5 Analytic and Translational Genetics Unit, Massachusetts General Hospital, Boston, MA 02114 USA

## Abstract

We summarize the remarkable progress that has been made in the identification and functional characterization of DNA sequence variants associated with disease.

## Editorial

The central objectives of human genetic research are to identify the sequence variation that plays a causal role in the development of disease, and then to use this information to generate insights into the biology of health and disease that can support clinical translation. These objectives have long been realized for a subset of diseases attributable to rare, high-penetrance alleles but, until recently, the challenges inherent in extending this success to the common, multifactorial diseases that account for most human illness have often seemed insurmountable. Nevertheless, over the past decade, the advent of genome-scale approaches for testing variant association to disease, and their application to increasingly large sample sets, has transformed our ability to identify alleles underlying rare and common diseases alike. At the same time, the arrival of a growing battery of sequence-based genomic assays has accelerated the high-throughput characterization of variant and transcript function, and these approaches are increasingly able to highlight the mechanisms through which disease-risk alleles operate. This is the best of times for human disease genomics, and this special issue of *Genome Biology* charts the remarkable progress that the community has made.

The first theme that emerges from the manuscripts published in this issue is the tremendous progress now being made in understanding the genetic basis of rare, typically monogenic, diseases, thanks in large part to rapid advances in the development and uptake of high-throughput DNA-sequencing methods. The advent of cost-effective exome sequencing in particular has ushered in a second golden age of Mendelian disease gene discovery, in large part due to the ability to discover causal variants that were inaccessible with previous technologies. This includes a wave of discovery of newly occurring (de novo) mutations that were largely invisible in the era of family linkage studies [1], as well as the detection of complex structural rearrangements that were difficult or impossible to characterize with previous array-based methods [2]. In this issue, authors report the highly successful application of large-scale sequencing approaches to the diagnosis of ciliopathies [3] and disorders of sexual development [4], and to the discovery, through linkage and exome sequencing, of a novel gene for a complex neuropsychiatric disorder [5].

One of the crucial challenges in the genomic era of rare disease diagnosis is determining exactly which of the many potentially functional variants found in any patient’s genome actually contributes to their disease. This process is complicated by the fact that existing databases of reported disease-causing mutations are heavily contaminated by false-positive reports of pathogenicity, in large part a hangover from an era of discovery in the absence of large databases of variant frequency in the general population. In this issue, Abouelhoda and colleagues [6] demonstrate that exome-sequencing data from Saudi Arabian samples can help reclassify many such variants, due to the largely unexplored nature of the variation in the Middle Eastern region and the presence of autozygosity. Nevertheless, much work remains to be done to clean up variant databases and to empower accurate variant classification as we move into an era of increasingly pervasive sequencing in both disease patients and healthy individuals.

The second theme that emerges is the increasing recognition that monogenic and complex disease are not discrete entities, but rather lie along a general spectrum of human disease. For a growing number of alleles implicated in monogenic disease, wider access to sequence data has revealed the extent to which ascertainment through densely affected pedigrees has led to a general overestimation of penetrance. Genotypes previously thought to be tightly coupled to significant early-onset pathology have often turned out to be compatible with normal health. In some cases, the variants are genuinely innocuous, and there has been false attribution of their relationship to disease. In others, the variants concerned have variable penetrance, presumably reflecting the contribution of other genetic, environmental, or stochastic factors that modulate their phenotypic impact. From the perspective of common disease research, there are ever more examples of genes that harbor an extended series of disease-associated alleles, ranging widely in allele frequency and effect size. In this issue, for instance, Jansen and colleagues [7] describe how whole-exome sequencing in unrelated subjects with non-familial Parkinson’s disease has revealed multiple genes that contain homozygous (or compound heterozygous) loss-of-function alleles, several of which displayed disease-relevant phenotypes when manipulated in appropriate cellular models.

The historical distinction between ‘Mendelian’ and ‘polygenic’ alleles is a throwback to 19th century arguments between the Mendelian and biometrician schools of evolutionary thought, a debate sustained into the modern era by the restricted discovery range of the earliest genome-wide techniques—linkage analysis in multiplex pedigrees on the one hand, common variant genome-wide association analysis on the other. With sequence-based discovery now possible across a variety of sample types—from pedigrees and trios to unselected populations—the continuum of risk allele effects and frequencies has been exposed, and can be exploited for mechanistic and translational benefit [8]. In this issue, Delahaye-Duriez and colleagues [9] demonstrate how sets of genes implicated in both rare and common forms of epilepsy converge on a pathway of proteins involved in synaptic function, and Yu and colleagues [10] show that a set of genes in which sequence variants are associated with serum amino acid levels overlap strikingly with known Mendelian metabolic disease loci. A detailed understanding of both rare, early-onset disease and common, later-onset disease will require a more holistic view of genetic risk. As outlined in two other contributions, the phenotypic consequences of sequence variation can not only be observed at the level of the individual, but also play out over many generations through their impact on fecundity, as revealed through evidence of selection [11, 12].

The identification of risk-alleles is a largely sterile exercise until it is possible to use them to drive improvements in the understanding of disease pathogenesis. The third theme represented in the manuscripts published in this special issue is the accelerating pace with which the integration of genome-scale annotation is delivering biological meaning for the discovered alleles. For many common diseases, it is becoming increasingly clear that most of the genetic variance is attributable to common variants. The variants responsible overwhelmingly map in non-coding sequence, and presumably act through the transcriptional regulation of downstream effector transcripts, often in tissue-specific ways.

The advent of high-throughput genome-scale technologies for mapping sites of regulatory impact (through assays of chromatin accessibility and modification, methylation status, transcription factor binding, and the like) and their implementation across a wide range of tissues (pioneered by the ENCODE consortium and the Epigenome Road Map) has provided the Rosetta Stone that links risk variant localization to functional impact. The integration of genome-wide genetic association data with genomic-scale annotation data has often resulted in a virtuous cycle of mutual advantage, defining the tissues, and sometimes the specific cell types, that are central to the pathogenesis of the disease of interest, supporting more accurate fine-mapping of risk alleles and characterizing key regulatory circuitry. Papers in this issue provide several examples, including studies in schizophrenia [13] and in inflammatory conditions [14, 15].

In addition, by linking regulatory elements to their downstream targets, for example through detection of *cis*-expression signals [16] and physical DNA–DNA interactions [17], researchers have been able to make more confident assignments regarding the effector transcripts through which the various risk-alleles exert their phenotypic impact. Additional insights have flowed from the ability to compare the genome-wide association profiles of apparently diverse phenotypes, highlighting unsuspected overlaps, for example between the innate immune system and autism spectrum disorder [18]. Key to these advances has been the development of novel tools for the statistical analysis and visualization of these complex data sets; two such tools are described in this issue [19, 20].

The next few years will bring increasingly massive genomic data sets from patients and controls, as well as more sophisticated mechanisms for the analysis and integration of a wide variety of genomic data types. We can expect to see not only continued growth in the number of disease genes identified, but also a deepening of our understanding of the fundamental genetic architecture of human disease states, and a transformation in our ability to move from associated genes, to pathways, to biology and clinical translation. The articles in this special issue illustrate our community’s exciting progress along each of these avenues.
